# High-Accuracy Approximation of High-Rank Derivatives: Isotropic Finite Differences Based on Lattice-Boltzmann Stencils

**DOI:** 10.1155/2014/142907

**Published:** 2014-01-29

**Authors:** Keijo Kalervo Mattila, Luiz Adolfo Hegele Júnior, Paulo Cesar Philippi

**Affiliations:** ^1^Laboratory of Porous Media and Thermophysical Properties, Mechanical Engineering Department, Federal University of Santa Catarina, 88040-900 Florianópolis, SC, Brazil; ^2^Department of Petroleum Engineering, State University of Santa Catarina, 88330-668 Balneário Camboriú, SC, Brazil

## Abstract

We propose isotropic finite differences for high-accuracy approximation of high-rank derivatives. These finite differences are based on direct application of lattice-Boltzmann stencils. The presented finite-difference expressions are valid in any dimension, particularly in two and three dimensions, and any lattice-Boltzmann stencil isotropic enough can be utilized. A theoretical basis for the proposed utilization of lattice-Boltzmann stencils in the approximation of high-rank derivatives is established. In particular, the isotropy and accuracy properties of the proposed approximations are derived directly from this basis. Furthermore, in this formal development, we extend the theory of Hermite polynomial tensors in the case of discrete spaces and present expressions for the discrete inner products between monomials and Hermite polynomial tensors. In addition, we prove an equivalency between two approaches for constructing lattice-Boltzmann stencils. For the numerical verification of the presented finite differences, we introduce 5th-, 6th-, and 8th-order two-dimensional lattice-Boltzmann stencils.

## 1. Introduction

The approximation of derivatives by finite differences is the cornerstone of numerical computing. Forward, backward, and central differences, the five-point stencil for approximating Laplacian in a two-dimensional domain, and the numerical analysis of the convergence rate of the related approximation errors, based on the application of Taylor series, require no introduction for anyone working in the field of scientific computing.

Construction of finite-difference stencils for the approximation of high-rank derivatives in two or three dimensions, say gradient of Laplacian or biLaplacian, will already be a more advanced topic—even if achieved by solving a modest linear system of equations. A further complication is introduced when requiring an isotropic approximation of derivatives. More specifically, when the leading-order error term of the finite difference approximation is required to be an isotropic expression or, in other words, to be free of directional bias. Such a property may be essential, for example, when solving certain partial differential equations.

Conventional finite differences are not isotropic in the above sense. Isotropic finite differences of second-order accuracy for the approximation of first and second derivatives, both in two and three dimensions, together with a systematic procedure for constructing the differences, are presented in [1]. Patra and Karttunen proceed further: they present up to fourth-order accurate isotropic stencils, in two and three dimensions, for the numerical computation of second, third, and fourth derivatives [2].

In the context of lattice Boltzmann methods, isotropic finite differences have been well known for some time, mainly because of their importance in the approximation of interparticle forces in multiphase and multicomponent models. For example, in the so-called Shan-Chen multiphase model [3], as was remarked by Yuan and Schaefer [4], the originally proposed approximation of interparticle forces includes an isotropic finite-difference approximation of the gradient of the interaction potential. In fact, when the standard D2Q9 lattice-Boltzmann stencil is used, the approximation is equivalent to the one proposed by Kumar [1].

Recently new efforts were undertaken to construct, or define, isotropic finite-difference stencils by utilizing the lattice-Boltzmann method framework. Namely, in the construction of lattice-Boltzmann stencils, a set of weights and discrete velocity vectors have to be found, together with a scaling factor related to the lattice speed of sound, in such a way that these weights and velocities will fulfill isotropy conditions up to a given order—a property ensuring a correspondence between continuous and discrete moments of the equilibrium distribution functions [5–7]. For example, in [8–10], this construction procedure is adopted in order to produce isotropic finite-difference stencils: there second-order accurate approximations are presented, but the isotropy goes beyond the leading order error term.

Lee et al. present a different strategy: they propose to approximate derivatives in 2D and 3D by taking moments of the conventional 1D finite differences along the characteristic lines—the moments are isotropic finite-differences [11, 12]. In the computation of these moments, the weights, discrete velocity vectors, and the scaling factor of a lattice Boltzmann stencil are utilized. Philippi et al. present up to fourth-order accurate isotropic finite difference stencils, constructed directly with the weights and velocity vectors of a given lattice Boltzmann stencil, for the approximation of gradient, Laplacian, and gradient of Laplacian [13]. Independently of Philippi et al., but by adopting the same approach, second-order accurate isotropic finite-difference approximation of Laplacian was proposed by Thampi et al. [14]. In [15], following the work of Thampi et al., approximations for the divergence and curl were presented together with fourth-order approximations for the gradient and Laplacian.

Naturally, in both approaches by Lee and Philippi, the validity of the presented expressions for the isotropic finite differences, and their isotropy properties, depends directly on the order of isotropy fulfilled by the lattice Boltzmann stencil utilized. On the other hand, these approaches provide beauty in abstraction: expressions for the finite differences are valid regardless of dimension, and any lattice Boltzmann stencil isotropic enough can be utilized—a family of finite differences is defined with a single expression.

Here our purpose is to go further by presenting isotropic finite differences, based on the direct utilization of lattice Boltzmann stencils, for the high-accuracy approximation of high-rank derivatives. In the construction of these isotropic differences we make use of the Hermite polynomial tensors in a very similar manner in which they are used in the construction of so-called kinetic projectors. Theory of Hermite polynomial tensors was first introduced by Grad in his innovative article [16]. This formal approach allows us to have very compact and abstract expressions for the coefficients of the stencils. It also allows the isotropy and accuracy properties of the proposed approximations to be derived theoretically. After these formal developments, we resort to simple calculus and construct, hierarchically, higher-order accurate stencils for the approximation of high-rank derivatives.

First, an introduction to Hermite polynomial tensors, monomials and their properties is presented in Section 2 using a specific notation. The fundamentals of lattice-Boltzmann schemes and stencils are then introduced in Section 3. Also in this section, 5th-, 6th-, and 8th-order lattice-Boltzmann stencils for two dimensions are introduced. The theory of using Hermite polynomial tensors together with the weights, velocity vectors, and the scaling factor for the construction of isotropic differences is presented in Section 4. This is followed by the hierarchical calculus of higher-order accurate stencils in Section 5. Application of the proposed stencils is discussed in Section 6. The discussion involves explicit expressions for the coefficients of some finite-difference approximations; these coefficients are then compared with other corresponding approximations proposed in the literature. The new lattice-Boltzmann stencils, introduced in Section 3, are utilized in Section 7 for the numerical verification of the proposed finite differences. Finally, a short conclusion is presented.

## 2. Theoretical Background

The theory of lattice-Boltzmann method, as well as the theory of isotropic finite differences proposed in this work, relies on Hermite polynomial tensors, monomials, and their properties. In this section, these fundamental concepts are defined using a specific notation explained below. This section is strongly based on the work of Grad [16].

### 2.1. Notational Conventions

The presentation of explicit expressions for Hermite polynomial tensors, or the related mathematical derivations, in an unambiguous yet comprehensible way is not an easy task because the expressions involve combinations. Here we will adopt the notation used by Grad in his original article with a minor modification. Namely, let the nonstandard operation

(1) $$\begin{array}{c} A_{\alpha_{1}\cdots \alpha_{k}}^{\left(k\right)}⊛B_{\beta_{1}\cdots \beta_{n-k}}^{\left(n-k\right)}\equiv A^{\left(k\right)}⊛B^{\left(n-k\right)} \end{array}$$

denote summation of tensor products over all possible combinations of indices: the number of combinations is *n*!/(*k*!  (*n* − *k*)!), where *n* is the rank of the tensor products. For example,

(2) A(2)⊛B(2)≡Aαβ(2)⊛Bγδ(2)=Aαβ(2)Bγδ(2)+Aαγ(2)Bβδ(2)+Aαδ(2)Bβγ(2)+Aβγ(2)Bαδ(2)+Aβδ(2)Bαγ(2)+Aγδ(2)Bαβ(2).

The notation is a shorthand, and it always has precedence over all other mathematical operations and manipulations. A similar notation was recently adopted in [17] for the same purpose of facilitating mathematical treatments.

Furthermore, the subscripts with bold typesetting, **p** and **q**, denote separate nonempty index sets; that is, **p** = {*α*_1_ ⋯ *α*_*k*_} and **q** = {*β*_1_ ⋯ *β*_*l*_}; **p** **q** denotes their union and the number of indices in each set is implied by the context. The operator ⊛ is used in conjunction with these index sets: the implied summations over index combinations are only over the shared index set **q**—the index set **p** is considered as fixed. For example, when **p** = {*α*} and **q** = {*βγδ*},

(3) $$\begin{array}{c} A_{pq}^{\left(2\right)}⊛B_{q}^{\left(2\right)}=A_{\alpha \beta }^{\left(2\right)}B_{\gamma \delta }^{\left(2\right)}+A_{\alpha \gamma }^{\left(2\right)}B_{\beta \delta }^{\left(2\right)}+A_{\alpha \delta }^{\left(2\right)}B_{\beta \gamma }^{\left(2\right)} \end{array}$$

or when **p** = {*α*} and **q** = {*βγδ* *ϵ*},

(4) Apq(3)⊛Bq(2)=Aαβγ(3)Bδϵ(2)+Aαβδ(3)Bγϵ(2)+Aαβϵ(3)Bγδ(2) +Aαγδ(3)Bβϵ(2)+Aαγϵ(3)Bβδ(2)+Aαδϵ(3)Bβγ(2).

The appendix of [6] provides an alternative notation for the same operations.

With Δ^(*n*)^ ≡ Δ_*α*_1_⋯*α*_2*n*__^(*n*)^ we denote the generalized Kronecker delta: these isotropic tensors are symmetric with respect to all of their 2*n* subscripts. The first generalized Kronecker deltas are

(5) Δ(0)=1,Δαβ(1)=δαβ,Δαβγδ(2)=(δαβδγδ+δαγδβδ+δαδδβγ),Δαβγδϵζ(3)=(δαβΔγδϵζ(2)+δαγΔβδϵζ(2)+δαδΔβγϵζ(2)+δαϵΔβγδζ(2)+δαζΔβγδϵ(2)),Δαβγδϵζηθ(4)=(δαβΔγδϵζηθ(3)+δαγΔβδϵζηθ(3)+δαδΔβγϵζηθ(3)+δαϵΔβγδζηθ(3)+δαζΔβγδϵηθ(3)+δαηΔβγδϵζθ(3)+δαθΔβγδϵζη(3)).

Their expressions are given by the recurrence relation

(6) $$\begin{array}{c} \Delta_{pq}^{\left(n\right)}=\Delta_{pq}^{\left(1\right)}⊛\Delta_{q}^{\left(n-1\right)}. \end{array}$$

The number of separate terms in the expressions for the generalized Kronecker delta, if written only by using the standard Kronecker delta symbol, is given by the so-called double factorial (2*n*)!/(2^*n*^ *n*!). For example, Δ^(4)^ and Δ^(8)^ involve 105 and 2 027 025 separate terms, respectively.

In addition, by Λ^(*n*)^ ≡ Λ_*α*_1_⋯*α*_*n*_*β*_1_⋯*β*_*n*__^(*n*)^ we denote rank 2*n* orthogonality tensor. The first orthogonality tensors are

(7) Λ(0)=1,Λα1β1(1)=δα1β1,Λα1α2β1β2(2)=(δα1β1δα2β2+δα1β2δα2β1),Λα1α2α3β1β2β3(3)=(δα1β1δα2β2δα3β3+δα1β1δα2β3δα3β2 +δα1β2δα2β1δα3β3+δα1β2δα2β3δα3β1 +δα1β3δα2β1δα3β2+δα1β3δα2β2δα3β1).

More generally, Λ^(*n*)^ is a sum of *n*! terms where each term is a product of *n* Kronecker deltas; the sum is over permutation of the indices so that each Kronecker delta has always one index from the set *α*_*k*_ and the other from *β*_*l*_. The orthogonality tensors can be defined by the recurrence relation

(8) $$\begin{array}{c} \Lambda_{\alpha_{1}\cdots \alpha_{n}q}^{\left(n\right)}=\Delta_{\alpha_{1}q}^{\left(1\right)}⊛\Lambda_{\alpha_{2}\cdots \alpha_{n}q}^{\left(n-1\right)}. \end{array}$$

The tensor Λ^(*n*)^ can also be regarded as a symmetry operator. That is, a tensor product between an arbitrary rank *n* tensor and Λ^(*n*)^ will extract the symmetric part of that tensor. When the arbitrary tensor **A** ^(*n*)^ is already symmetric, the product is (Einstein summation convention is applied)

(9) $$\begin{array}{c} \Lambda_{\alpha_{1}\cdots \alpha_{n}\beta_{1}\cdots \beta_{n}}^{\left(n\right)}A_{\beta_{1}\cdots \beta_{n}}^{\left(n\right)}=n!A_{\alpha_{1}\cdots \alpha_{n}}^{\left(n\right)}. \end{array}$$

Finally, a summation over index combinations can be split into two separate summations. Let **A** ^(*k*)^ and **B** ^(*m*)^ again denote arbitrary tensors, and **p** is assumed to be a single index set. Then,

(10) $$\begin{array}{c} {\left(A^{\left(k\right)}⊛B^{\left(m\right)}\right)}_{pq}=A_{pq}^{\left(k\right)}⊛B_{q}^{\left(m\right)}+B_{pq}^{\left(m\right)}⊛A_{q}^{\left(k\right)}, \end{array}$$

where the left-hand side involves all possible index combinations from the set **p** **q**.

### 2.2. Hermite Polynomial Tensors, Monomials, and Their Properties

Let us define a monomial tensor **M** ^(*n*)^(**v**) ≡ *ℳ*_*α*_1_⋯*α*_*n*__^(*n*)^ : = *v*_*α*_1__ ⋯ *v*_*α*_*n*__; notation **M** ^(*n*)^ will always imply an argument **v**. Hermite polynomial tensors **H** ^(*n*)^ ≡ *ℋ*_*α*_1_⋯*α*_*n*__^(*n*)^ are defined by the formula

(11) $$\begin{array}{c} H^{\left(n\right)}\left(v\right)=\frac{{\left(-1\right)}^{n}}{\omega \left(v\right)}\nabla_{v}^{\left(n\right)}\omega \left(v\right),\\ \omega \left(v\right)=\frac{1}{{\left(2\pi \right)}^{D/2}\;\;}e^{-(v^{2}/2)},\;\nabla_{v}^{\left(n\right)}\equiv \partial_{\alpha_{1}}\cdots \partial_{\alpha_{n}}, \end{array}$$

where *ω* is a weighting function, *D* refers to the spatial dimension, and *v* ^2^ = *v*_*α*_ *v*_*α*_. Like with monomials, notation **H** ^(*n*)^ will always imply an argument **v**. Furthermore, a rank *n* Hermite polynomial tensor is also a polynomial of degree *n*. The explicit expressions for the first few Hermite polynomial tensors are given in Appendix A.

An important property related to the weighting function is

(12) $$\begin{array}{c} \int \omega \left(v\right)M^{\left(2n\right)}dv=\Delta^{\left(n\right)}. \end{array}$$

Equation (12) defines the moments of the weighting function; all odd order moments vanish. Clearly, (12) can also be regarded as an expression for the weighted tensorial inner product of two monomials. An analogous expression for the inner product of Hermite polynomial tensors is

(13) ∫ω(v)H(n)H(m)dv≡∫ω(v)Hα1⋯αn(n)Hβ1⋯βm(m)dv={0,n≠m,Λ(n),n=m.

This means that not only Hermite polynomial tensors of different rank are orthogonal, but also distinct polynomials of the same degree.

In fact, an explicit formula for Hermite polynomial tensors is given by a simple summation:

(14) $$\begin{array}{c} H^{\left(2n+s\right)}=\sum_{k=0}^{n}{\left(-1\right)}^{k}M^{\left(2(n-k)+s\right)}⊛\Delta^{\left(k\right)}, \end{array}$$

where *s* = 0 and *s* = 1 define even and odd tensors, respectively. A useful recurrence relation for Hermite polynomial tensors is

(15) $$\begin{array}{c} v_{p}H_{q}^{\left(n\right)}=H_{pq}^{\left(n+1\right)}+\Delta_{pq}^{\left(1\right)}⊛H_{q}^{\left(n-1\right)}. \end{array}$$

The recurrence relation is very convenient for a computer implementation of high-rank Hermite polynomial tensors. Useful mathematical tools are provided also by the two relations

(16) $$\begin{array}{c} \nabla_{p}^{\left(1\right)}H_{q}^{\left(n\right)}=\Delta_{pq}^{\left(1\right)}⊛H_{q}^{\left(n-1\right)},\;\;\;\nabla_{p}^{\left(1\right)}M_{q}^{\left(n\right)}=\Delta_{pq}^{\left(1\right)}⊛M_{q}^{\left(n-1\right)}. \end{array}$$

### 2.3. Inner Products in Discrete Spaces

Above we presented weighted tensorial inner products between two monomials and between two Hermite polynomial tensors in a continuous space. However, numerical methods inherently involve discrete spaces. Hence, it is of computational interest to construct discrete representations for the continuous space admitting the above inner products. The construction of such a discrete representation is basically a quadrature problem: find the discrete weights *w*_*i*_ and abscissas **v**_*i*_ so that

(17) $$\begin{array}{c} \sum_{i}w_{i}M_{i}^{\left(2n\right)}=\Delta^{\left(n\right)}=\int \omega \left(v\right)M^{\left(2n\right)}dv \end{array}$$

or

(18) $$\begin{array}{c} \sum_{i}w_{i}H_{i}^{\left(n\right)}H_{i}^{\left(n\right)}=\Lambda^{\left(n\right)}=\int \omega \left(v\right)H^{\left(n\right)}H^{\left(n\right)}dv \end{array}$$

is satisfied up to a given order *N*; in the general solution *w*_*i*_ ≠ *ω*(**v**_*i*_). In fact, in Appendix B we prove that a solution to either of the two quadrature problems will guarantee both (17) and (18).

Furthermore, matching the inner products of two Hermite polynomial tensors of equal rank, up to a given order *N*, will guarantee orthogonality of the Hermite polynomial tensors in the discrete space:

(19) $$\begin{array}{c} \sum_{i}w_{i}H_{i}^{\left(n\right)}H_{i}^{\left(m\right)}=\{\begin{array}{cc} 0, & n\neq m,\\ \Lambda^{\left(n\right)}, & n=m, \end{array}\;n+m\leq 2N+1. \end{array}$$

In other words, the inner products of two Hermite polynomial tensors, not necessarily of equal rank, will match in the discrete and continuous spaces. This has been shown in the appendix of [5].

Now we will proceed to the discrete inner product between a monomial and a Hermite polynomial tensor. In Appendix A, explicit expressions are given for the first few monomials as linear combinations of Hermite polynomial tensors. In fact, these expressions are given by a simple summation formula:

(20) $$\begin{array}{c} M^{\left(2n+s\right)}=\sum_{k=0}^{n}H^{\left(2(n-k)+s\right)}⊛\Delta^{\left(k\right)}; \end{array}$$

even and odd tensors are defined by *s* = 0 and *s* = 1, respectively. A proof for this summation formula is given in Appendix C. Since the Hermite polynomial tensor expansion for the monomials is now available, we find that

(21) $$\begin{array}{c} \sum_{i}w_{i}H_{i}^{\left(n\right)}M_{i}^{\left(m\right)}=\sum_{i}w_{i}H_{i}^{\left(n\right)}H_{i}^{\left(m\right)},\;m\leq n\leq N, \end{array}$$

(22) $$\begin{array}{c} \sum_{i}w_{i}H_{i,p}^{\left(n\right)}M_{i,q}^{\left(n+2k\right)}=\Lambda_{pq}^{\left(n\right)}⊛\Delta_{q}^{\left(k\right)},\;k\geq 1. \end{array}$$

Inner products between **H**_*i*_^(*n*)^ and **M**_*i*_^(*n*+2*k*+1)^, *k* ≥ 0, vanish. Note that (22) is valid only when (*n* + *k*) ≤ *N*.

## 3. Lattice-Boltzmann Method

The principal variable in Boltzmann model equations is the mass distribution function *f*(**r**, *t*, **c**): the arguments **r**, *t*, and **c** refer to the spatial, temporal, and microscopic velocity space, respectively. Lattice-Boltzmann methods can be directly derived from Boltzmann model equations. The first step in the derivation is to discretize the microscopic velocity space **c** = *c*_*T*_ **v**, where **v** is a dimensionless velocity, $c_{T}=\sqrt{k_{b}T_{0}/m}$ the thermal reference velocity, *k*_*b*_ the Boltzmann constant, *m* the molecular mass of the fluid, and *T*_0_ a reference temperature. The relevance of Hermite polynomial tensors and monomials to lattice-Boltzmann method is related to the discretization of the velocity space.

Particularly, in conventional lattice-Boltzmann schemes, the discrete velocity vectors always connect two sites of a uniform lattice; that is, (**r** + *δ*_*t*_ **c**_*i*_) is a lattice site whenever **r** is. By *h*_*r*_ and *δ*_*t*_ we denote the spatial spacing of a uniform lattice and the discrete time step of the temporal evolution, respectively; the lattice reference velocity *c*_*r*_ = *h*_*r*_/*δ*_*t*_. It is hence implied that **c**_*i*_ = *c*_*r*_ **c**_*i*_^⋆^ = *c*_*T*_ **v**_*i*_, where **c**_*i*_^⋆^ denote appropriate dimensionless discrete velocities. The relation *c*_*r*_/*c*_*T*_ = *a*_*s*_ is called the scaling factor. The triplet **c**_*i*_, *w*_*i*_, and *a*_*s*_ is called a lattice-Boltzmann stencil.

A way to construct lattice-Boltzmann stencils is to first prescribe **c**_*i*_^⋆^ and to assign **v**_*i*_ = *a*_*s*_ **c**_*i*_^⋆^. Moreover, the discrete velocity set is prescribed so that a vector from the set always has an opposite counterpart; that is, **c**_−*i*_ = −**c**_*i*_ where index −*i* refers to the opposite vector—the zero or rest vectors are an obvious exception. The unknowns *a*_*s*_ and *w*_*i*_ are then defined by solving either of the two quadrature problems, (17) or (18), up to a given order *N*. This is the method of prescribed abscissas [5]. The underlying motivation is to ensure correspondence between continuous and discrete moments of the equilibrium distribution functions. For example, the well-known stencils D2Q9 and D2V37 are of second and fourth order (*N* = 2 and *N* = 4) [5, 18]. Specification of three lattice-Boltzmann stencils are given in Tables 1 and 2. The two-dimensional stencils are here presented for the first time: D2V49, D2V81, and D2V141 are fifth-, sixth-, and and eighth-order stencils, respectively. In Section 7 we will apply D2V141 to numerically confirm accuracy of our isotropic finite differences. Furthermore, D2V49 and D2V81 will be used in a numerical comparison of some specific finite-difference approximations.

The simplest evolution equation, that is, a lattice-Boltzmann equation (LBE), for the single-particle distribution function *f*_*i*_(**r**, *t*) ≡ *f*(**r**, *t*, **c**_*i*_) is

(23) $$\begin{array}{c} f_{i}\left(r+\delta_{t}c_{i},t+\delta_{t}\right)=f_{i}\left(r,t\right)+\delta_{t}\Omega_{i}\left(r,t\right),\;i=0,\ldots ,q-1, \end{array}$$

where *Ω*_*i*_ is, in general, a nonlinear collision operator. For example, *Ω*_*i*_ = −*f*_*i*_^neq^/*τ* will specify the famous lattice-BGK equation involving only a single relaxation time *τ*. The nonequilibrium function *f*_*i*_^neq^ : = *f*_*i*_ − *f*_*i*_^eq^ is defined with respect to the equilibrium function *f*_*i*_^eq^ discussed below. The equilibrium function, in turn, is defined by the conserved hydrodynamic variables, that is, by the first few moments of the distribution function in the velocity space:

(24) $$\begin{array}{c} \rho =\sum_{i}f_{i},\;\;\rho u=\sum_{i}f_{i}c_{i},\;\;\rho \epsilon =\sum_{i}f_{i}{\left(c_{i}-u\right)}^{2}; \end{array}$$

the local fluid density *ρ*, velocity **u**, and internal energy *ϵ* are the zeroth, first, and second moment of the distribution function, respectively. The above LBE results from a first-order discretization along the characteristics: higher-order discretizations are considered, for example, in [19].

A discrete equilibrium function can be specified by expanding the famous Maxwell-Boltzmann equilibrium distribution function in Hermite polynomial tensors; the expansion is truncated to a given order. For isothermal models, the resulting discrete equilibrium function is

(25) fieq(ρ,u)=ρ(𝒦i(0)+𝒦i,α(1)uα+𝒦i,αβ(2)uαuβ +𝒦i,αβγ(3)uαuβuγ+⋯),

where

(26) $$\begin{array}{c} {𝒦}_{i,\alpha_{1}\cdots \alpha_{n}}^{\left(n\right)}\equiv K_{i}^{\left(n\right)}:=\frac{w_{i}}{n!c_{T}^{2n}}H^{\left(n\right)}\left(c_{i}\right) \end{array}$$

is the so-called kinetic projector mapping hydrodynamic variables into a space of kinetic description; note the relation **H** ^(*n*)^(**c**_*i*_) = *c*_*T*_^*n*^ **H** ^(*n*)^(**v**_*i*_). In the next section we will base our isotropic finite-difference stencils on *difference projectors*: close analogs to kinetic projectors. From programming point of view, there remain three properties which deserve a note.

1. By definition, Hermite polynomial tensors **H**_*i*_^(*n*)^ are symmetric in their *n* subscripts.
2. The expressions for even order Hermite polynomial tensors involve only even order monomials. Hence, **H**_−*i*_^(*n*)^ = **H**_*i*_^(*n*)^.
3. The expressions for odd order Hermite polynomial tensors involve only odd order monomials. Hence, **H**_−*i*_^(*n*)^ = −**H**_*i*_^(*n*)^.

By acknowledging these properties, whether implementing equilibrium functions based on kinetic projectors or isotropic finite differences based on difference projectors, a significant improvement in computational performance can be achieved. The number of independent components in a symmetric tensor can be obtained directly from *Pascal's triangle*. For example, a general rank-eight tensor, in three dimensions, has 3^8^ = 6 561 components whereas a symmetric tensor has only 45 independent components. Furthermore, the sum of components in general tensors up to rank eight, again in 3D, is 9 841; the corresponding number for symmetric tensors is 165—a number also directly obtained from Pascal's triangle. The second and third property together reduce the number of independent tensor components by a factor of two (but only approximately, because the discrete velocity set usually includes a zero vector, associated with the so-called rest particles, and it does not have an opposite counterpart).

## 4. Isotropic Finite Differences of Second-Order Accuracy

We propose to approximate partial derivatives of a function *g* with a simple finite difference:

(27) $$\begin{array}{c} \nabla^{\left(n\right)}g\left(r\right)\approx \sum_{i}D_{i}^{\left(n,m\right)}g\left(r+\delta_{t}c_{i}\right), \end{array}$$

where the rank *n* tensor **D**_*i*_^(*n*,*m*)^(**c**_*i*_) ≡ **D**_*i*_^(*n*,*m*)^ is the difference projector storing the coefficients of the finite-difference stencil; the superscript *m* denotes the order of accuracy of the finite difference. The second-order accurate difference projectors are the Hermite polynomial tensors multiplied by appropriate constants:

(28) $$\begin{array}{c} D_{i}^{\left(n,2\right)}\left(c_{i}\right):=\frac{w_{i}}{\delta_{t}^{n}c_{T}^{2n}}H^{\left(n\right)}\left(c_{i}\right), \end{array}$$

where *n* ≤ *N* and *N* is the order of the lattice-Boltzmann stencil.

In order to prove the second-order accuracy of **D**_*i*_^(*n*,2)^, we first Taylor-expand function *g* at the right-hand side of (27), use the definition in (28), and change the order of summation:

(29) ∑iDi,p(n,2)g(r+δtci) =1δtncT2n(∑m=0∞δtmm!∇q(m)∑iwiHp(n)(ci)Mq(m)(ci))g(r).

Note that here the repeated index set **q** emphasizes a full tensor contraction between **M**_*i*,**q**_^(*m*)^ and ∇_**q**_^(*m*)^. By using the inner products presented in Section 2.3 and by acknowledging the simple relations **H** ^(*n*)^(**c**_*i*_) = *c*_*T*_^*n*^ **H** ^(*n*)^(**v**_*i*_) and **M** ^(*m*)^(**c**_*i*_) = *c*_*T*_^*m*^ **M** ^(*m*)^(**v**_*i*_), we can transform the right-hand side into

(30) (1n!Λpq(n)∇q(n)+∑k=1N−nδt2kcT2k(n+2k)!∇q(n+2k) ×(Λpq(n)⊛Δq(k))+⋯)g(r).

Remember that the operator ⊛ has precedence over all other mathematical operations and manipulations. Hence, the correct interpretation above is to first expand the summation over combinations and then enforce the tensor contractions for each term separately.

Since ∇_**q**_^(*n*+2*k*)^ is clearly a symmetric tensor, we can make use of (9) and obtain Λ_**pq**_^(*n*)^∇_**q**_^(*n*+2*k*)^ = *n*!∇_**p**_^(*n*)^∇_**q**_^(2*k*)^, *k* ≥ 0; that is, the orthogonality tensor changes *n* indices from set **q** to set **p**. Furthermore, the contraction ∇_**q**_^(2*k*)^Δ_**q**_^(*k*)^ results in (∂^2^)^*k*^, where ∂^2^ = ∂_*α*_∂_*α*_ denotes Laplacian, (∂^2^)^2^ denotes Laplacian of Laplacian, and so on. In fact, because Δ_**q**_^(*k*)^ involves (2*k*)!/2^*k*^ *k*! terms, the Laplacians will be repeated an equal number of times. Finally, the summation over combinations includes (*n* + 2*k*)!/*n*!(2*k*)! terms. Hence

(31) ∑iDi,p(n,2)g(r+δtci) =∇p(n)(1+∑k=1N−nhr2kas2k  2k  k!(∂2)k+⋯)g(r).

This is our master equation. It confirms that the difference projectors **D**_*i*_^(*n*,2)^ defined in (28), together with the finite difference specified in (27), result in second-order approximations for the partial derivatives ∇^(*n*)^. Also, (31) exposes the isotropy: with **D**_*i*_^(*n*,2)^, the first (*N* − *n*) leading order error terms are isotropic (Laplacian is an isotropic operator).

## 5. High-Order Accurate Isotropic Finite Differences

After establishing the second-order accurate finite-difference stencils, we proceed to define higher-order accurate stencils. In order to do so, we explicitly write the first few leading-order error terms of the second-order approximation:

(32) ∑iDi(n,2)g(r+δtci) =∇(n)(1+hr22as2∂2+hr48as4(∂2)2+hr648as6(∂2)3+⋯)g(r).

With the above equation, it is straightforward to write higher-order difference projectors. First, the fourth-order accurate finite-difference stencils are defined with

(33) $$\begin{array}{c} D_{i,\alpha }^{\left(1,4\right)}=D_{i,\alpha }^{\left(1,2\right)}-\frac{h_{r}^{2}}{2a_{s}^{2}}D_{i,\alpha \beta \beta }^{\left(3,2\right)},\\ D_{i,\alpha \beta }^{\left(2,4\right)}=D_{i,\alpha \beta }^{\left(2,2\right)}-\frac{h_{r}^{2}}{2a_{s}^{2}}D_{i,\alpha \beta \gamma \gamma }^{\left(4,2\right)},\\ D_{i,\alpha \beta \gamma }^{\left(3,4\right)}=D_{i,\alpha \beta \gamma }^{\left(3,2\right)}-\frac{h_{r}^{2}}{2a_{s}^{2}}D_{i,\alpha \beta \gamma \delta \delta }^{\left(5,2\right)},\\ D_{i,\alpha \beta \gamma \delta }^{\left(4,4\right)}=D_{i,\alpha \beta \gamma \delta }^{\left(4,2\right)}-\frac{h_{r}^{2}}{2a_{s}^{2}}D_{i,\alpha \beta \gamma \delta \epsilon \epsilon }^{\left(6,2\right)},\\ D_{i,\alpha \beta \gamma \delta \epsilon }^{\left(5,4\right)}=D_{i,\alpha \beta \gamma \delta \epsilon }^{\left(5,2\right)}-\frac{h_{r}^{2}}{2a_{s}^{2}}D_{i,\alpha \beta \gamma \delta \epsilon \zeta \zeta }^{\left(7,2\right)},\\ D_{i,\alpha \beta \gamma \delta \epsilon \zeta }^{\left(6,4\right)}=D_{i,\alpha \beta \gamma \delta \epsilon \zeta }^{\left(6,2\right)}-\frac{h_{r}^{2}}{2a_{s}^{2}}D_{i,\alpha \beta \gamma \delta \epsilon \zeta \eta \eta }^{\left(8,2\right)}. \end{array}$$

Now by using the above fourth-order stencils, we can write the sixth-order stencils:

(34) $$\begin{array}{c} D_{i,\alpha }^{\left(1,6\right)}=D_{i,\alpha }^{\left(1,2\right)}-\frac{h_{r}^{2}}{2a_{s}^{2}}D_{i,\alpha \beta \beta }^{\left(3,4\right)}-\frac{h_{r}^{4}}{8a_{s}^{4}}D_{i,\alpha \beta \beta \gamma \gamma }^{\left(5,2\right)},\\ D_{i,\alpha \beta }^{\left(2,6\right)}=D_{i,\alpha \beta }^{\left(2,2\right)}-\frac{h_{r}^{2}}{2a_{s}^{2}}D_{i,\alpha \beta \gamma \gamma }^{\left(4,4\right)}-\frac{h_{r}^{4}}{8a_{s}^{4}}D_{i,\alpha \beta \gamma \gamma \delta \delta }^{\left(6,2\right)},\\ D_{i,\alpha \beta \gamma }^{\left(3,6\right)}=D_{i,\alpha \beta \gamma }^{\left(3,2\right)}-\frac{h_{r}^{2}}{2a_{s}^{2}}D_{i,\alpha \beta \gamma \delta \delta }^{\left(5,4\right)}-\frac{h_{r}^{4}}{8a_{s}^{4}}D_{i,\alpha \beta \gamma \delta \delta \epsilon \epsilon }^{\left(7,2\right)},\\ D_{i,\alpha \beta \gamma \delta }^{\left(4,6\right)}=D_{i,\alpha \beta \gamma \delta }^{\left(4,2\right)}-\frac{h_{r}^{2}}{2a_{s}^{2}}D_{i,\alpha \beta \gamma \delta \epsilon \epsilon }^{\left(6,4\right)}-\frac{h_{r}^{4}}{8a_{s}^{4}}D_{i,\alpha \beta \gamma \delta \epsilon \epsilon \zeta \zeta }^{\left(8,2\right)}. \end{array}$$

In a similar way, we use the sixth-order stencils in the definitions of eighth-order stencils:

(35) Di,α(1,8)=Di,α(1,2)−hr22as2Di,αββ(3,6)−hr48as4Di,αββγγ(5,4) −hr648as6Di,αββγγδδ(7,2),Di,αβ(2,8)=Di,αβ(2,2)−hr22as2Di,αβγγ(4,6)−hr48as4Di,αβγγδδ(6,4) −hr648as6Di,αβγγδδϵϵ(8,2).

Above we have defined the high-accuracy difference projectors in an hierarchical way. In practice, however, more straightforward definition might be more convenient for a computer implementation. That is, we can simply write out the expressions and get, for example,

(36) $$\begin{array}{c} D_{i,\alpha }^{\left(1,6\right)}=D_{i,\alpha }^{\left(1,2\right)}-\frac{h_{r}^{2}}{2a_{s}^{2}}D_{i,\alpha \beta \beta }^{\left(3,2\right)}+\frac{h_{r}^{4}}{8a_{s}^{4}}D_{i,\alpha \beta \beta \gamma \gamma }^{\left(5,2\right)},\\ D_{i,\alpha }^{\left(1,8\right)}=D_{i,\alpha }^{\left(1,2\right)}-\frac{h_{r}^{2}}{2a_{s}^{2}}D_{i,\alpha \beta \beta }^{\left(3,2\right)}+\frac{h_{r}^{4}}{8a_{s}^{4}}D_{i,\alpha \beta \beta \gamma \gamma }^{\left(5,2\right)}-\frac{h_{r}^{6}}{48a_{s}^{6}}D_{i,\alpha \beta \beta \gamma \gamma \delta \delta }^{\left(7,2\right)}. \end{array}$$

In this way, the high-accuracy projectors are defined using only second-order accurate projectors.

The degree of isotropy in the case of high-accuracy approximation of high-rank derivatives is discussed next. First we remind the reader that *n* denotes the rank of the derivative, *N* is the order of the lattice-Boltzmann stencil utilized, and *m* refers to the order of accuracy of the approximation. Then, the number of leading-order error terms which are isotropic, or the degree of isotropy, is

(37) $$\begin{array}{c} N_{I}=\left(N-n\right)-\left(m-2\right). \end{array}$$

## 6. Application of the Isotropic Finite Differences

In order to elucidate the usage of the above proposed finite-differences, we will provide the coefficients of some simple stencils in their most explicit form. Let us consider a particular application where the gradient and the gradient of Laplacian of an arbitrary function *g* must be approximated with fourth- and second-order accuracy, respectively. Furthermore, the approximations must be isotropic meaning that at least the leading-order error term is not directionally biased. In the case of the gradient, *n* = 1 and *m* = 4. Hence, from (37), *N* ≥ 4 in order to have *N*_*I*_ ≥ 1. The gradient of Laplacian case, *n* = 3 and *m* = 2, leads to the same conclusion. That is, at least a fourth-order lattice-Boltzmann stencil must be utilized: the well-known D2V37 stencil is a candidate [5].

Directly from (27) and (28), the second-order approximations of gradient and gradient of Laplacian, in their most explicit form, are

(38) $$\begin{array}{c} \partial_{\alpha }g\left(r\right)=\frac{a_{s}^{2}}{h_{r}}\sum_{i}w_{i}c_{i,\alpha }^{⋆}g\left(r+h_{r}c_{i}^{⋆}\right)+𝒪\left(h_{r}^{2}\right), \end{array}$$

(39) $$\begin{array}{c} \partial_{\alpha }\partial^{2}g\left(r\right)=\frac{a_{s}^{6}}{h_{r}^{3}}\sum_{i}w_{i}c_{i,\alpha }^{⋆}\left[c_{i}^{2}-\frac{B_{1}}{a_{s}^{2}}\right]g\left(r+h_{r}c_{i}^{⋆}\right)+𝒪\left(h_{r}^{2}\right), \end{array}$$

where *c*_*i*_^2^ ≡ *c*_*i*,*β*_^⋆^ *c*_*i*,*β*_^⋆^, the auxiliary coefficient *B*_1_ : = (*D* + 2), and *D* ≡ *δ*_*ββ*_. The approximation of the gradient, (38), is well known in the lattice-Boltzmann context and the approximation of the gradient of Laplacian, (39), is exactly the same as proposed in Appendix B of [13]. Furthermore, using the appropriate expression from Section 5, the explicit form of the fourth-order approximation of the gradient is

(40) $$\begin{array}{c} \partial_{\alpha }g\left(r\right)=\frac{a_{s}^{2}}{2h_{r}}\sum_{i}w_{i}c_{i,\alpha }^{⋆}\left[B_{2}-a_{s}^{2}c_{i}^{2}\right]g\left(r+h_{r}c_{i}^{⋆}\right)+𝒪\left(h_{r}^{4}\right), \end{array}$$

where *B*_2_ : = (*D* + 4). Again, the corresponding expression given by Philippi et al. agrees with this approximation (Equation (B1) in [13] involves a mistake: (40) is the correct expression).

Our approximations are derived from the theoretical basis developed in the previous sections. In [13], Philippi et al. use a three-step procedure for constructing their stencils: (1) the general function *g*(**r** + *δ*_*t*_ **c**_*i*_) is Taylor-expanded, (2) discrete moments (in the velocity space) of the Taylor expansion are computed, where the moments involve the discrete weights, and (3) linear combinations of these moments, and *g*(**r**), are computed so as to deliver appropriate approximations. That is, the three-step procedure allows tailored approximations which, in some cases, can be, for example, more compact than the approximations derived here. Thampi et al. proposed the same procedure [14], independently of Philippi et al., and applied it for a second-order accurate isotropic approximation of Laplacian.

As an example, let us consider second- and fourth-order accurate approximations of Laplacian. From (37), *N* ≥ 3 and *N* ≥ 5 in order to have *N*_*I*_ ≥ 1 for the two approximations, respectively. Hence, for example, the D2V17 and D2V49 stencils could be utilized. Using only (27) and (28), the explicit expressions for the second-order approximations of Laplacian an BiLaplacian (∂^2^∂^2^) are

(41) $$\begin{array}{c} \partial^{2}g\left(r\right)=\frac{a_{s}^{4}}{h_{r}^{2}}\sum_{i}w_{i}\left[c_{i}^{2}-\frac{D}{a_{s}^{2}}\right]g\left(r+h_{r}c_{i}^{⋆}\right)+𝒪\left(h_{r}^{2}\right), \end{array}$$

(42) ∂2∂2g(r)=as8hr4∑iwi[ci2(ci2−2as2B1)+Das4B1]×g(r+hrci⋆)+𝒪(hr2).

Then, with the appropriate expression from Section 5, the explicit form for the fourth-order approximation of the Laplacian is

(43) ∂2g(r)=as42hr2∑iwi[ci2(2B3−as2ci2)−Das2B2]×g(r+hrci⋆)+𝒪(hr4),

where *B*_3_ : = *D* + 3.

The corresponding second- and fourth-order approximations of Laplacian obtained with the three-step procedure, as proposed by Philippi et al., are

(44) $$\begin{array}{c} \partial^{2}g\left(r\right)=\frac{2a_{s}^{2}}{h_{r}^{2}}\left[\sum_{i}w_{i}g\left(r+h_{r}c_{i}^{⋆}\right)-g\left(r\right)\right]+𝒪\left(h_{r}^{2}\right), \end{array}$$

(45) ∂2g(r)=as2hr2[∑iwi(B2−as2ci2)g(r+hrci⋆)−4g(r)] +𝒪(hr4);

the same second-order approximation of Laplacian was also proposed by Thampi et al. [14]. In fact, (44) is equivalent to the approximation proposed earlier by Lee and Lin [11]. In addition, when (44) is utilized together with D2Q9, it coincides with the *Mehrstellen* approximation (see, e.g., [1, 2]).

Clearly, in the case of Laplacians, our approximations are not equivalent to those obtained using the three-step procedure. Moreover, the approximations given in (44) and (45) are isotropic whenever *N* ≥ 2 and *N* ≥ 4, respectively. Hence, for example, (45) allows more compact approximations than (43): the span (or the spatial extent in an axis direction) of the fifth-order stencil D2V49 presented in Table 1 is 5 lattice spacings, whereas the span of D2V37 is only 3.

Above the explicit forms of our approximations, still valid in any dimension, are given only as examples. In the implementations, however, it is not necessary to use the explicit forms in order to reach high computational efficiency; we consider it more convenient to use the abstract definitions given in the previous sections. First, even the high-rank Hermite polynomial tensors can be easily implemented, in an hierarchical manner, using the recurrence relation of (15). For example, preprocessor directives (macros), if available in the programming language, can be used for implementing the tensors. The finite-difference coefficients, that is, components of an appropriate difference projector, can then be stored by utilizing the appropriate Hermite polynomial tensors. Due to their definitions, the difference projectors share important properties with the Hermite polynomial tensors. Namely, the difference projectors are fully symmetric in their subscripts. Also,

(46) $$\begin{array}{c} D_{-i}^{\left(n,m\right)}=D_{i}^{\left(n,m\right)},\;\;D_{-i}^{\left(n,m\right)}=-D_{i}^{\left(n,m\right)} \end{array}$$

for even and odd *n*, respectively. Hence, a relatively small number of finite-difference coefficients need to be stored even when approximating high-rank derivatives.

## 7. Numerical Experiments and Discussion

In order to verify the accuracy of the presented finite differences, we numerically compute the derivative approximations and compare them against analytical solutions. Our analytical reference function is

(47) s(x,y,ω)=sin(ω  π(2x+y)),(x,y)∈[0,1]×[0,2], ω∈[1,2[,

for which derivatives, even high rank, are easily available. The additional constant parameter *ω* is introduced as an extra degree of freedom for the numerical experiments. In order to construct a square lattice (equal lattice spacing in the *x*- and *y*-directions), we always define the number of lattice nodes in the *y*-direction to be two times the number of nodes in the *x*-direction. Numerical error of the approximations is measured with a relative *L*_2_-error norm:

(48) $$\begin{array}{c} L_{2}=\sqrt{\frac{\sum ‍{\left(n\left(x,y\right)-a\left(x,y\right)\right)}^{2}}{\sum a{\left(x,y\right)}^{2}}}, \end{array}$$

where the summation is over all lattice nodes, *n*(*x*, *y*) denotes a numerically computed value, and *a*(*x*, *y*) is a corresponding analytical value.

**Figure 1:**
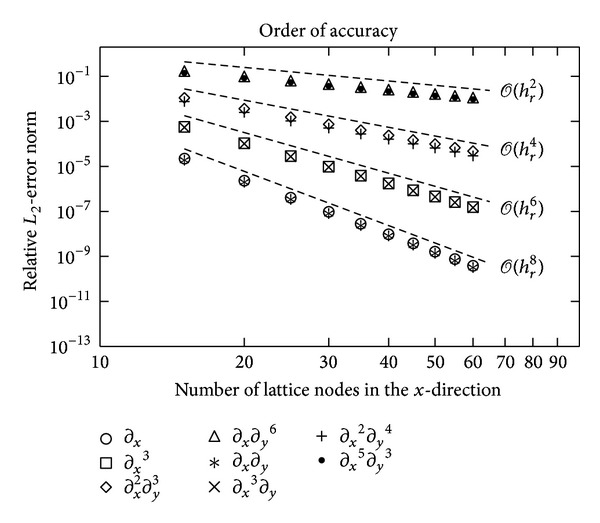
Order of convergence of the numerical error for some derivative approximations. The lattice-Boltzmann stencil D2V141 has been utilized. The dashed lines indicate 2nd-, 4th-, 6th-, and 8th-order convergence.

First we verify the theoretical convergence rates of the approximation errors. In such a verification, the truncation errors must dominate over the round-off errors arising from the floating-point arithmetic. Hence, we use relatively small lattices: in the reported results for the convergence rates, the number of nodes in the *x*-direction is between 15 and 60. Furthermore, we set *ω* = 1 and apply the eighth-order lattice-Boltzmann stencil D2V141, presented in Section 3, for the specification of the difference projectors. The difference projectors are defined in Sections 4 and 5.  Figure 1 reports the relative *L*_2_-error norms for some numerical approximations: for each rank, an approximation with a difference projector of highest attainable order of accuracy is chosen. Otherwise, the approximations for the figure are randomly chosen.  Figure 1 confirms the theoretical results for the chosen approximations; we have checked all the approximations and the theoretical results are indeed verified. In the approximations of high-rank derivatives, round-off errors start to dominate with larger lattice spacings than in the approximations of lower-rank derivatives—as expected.

Next we measure the computational efficiency of the approximations proposed here. For reference, we also measure the efficiency of some previously published approximations. In the approach of Lee et al. [11, 12], derivatives in 2D and 3D are approximated by taking moments of the conventional 1D finite differences along the characteristic lines. As an example, in order to have a minimal isotropic fourth-order approximation of the first derivative—only the leading-order error term is isotropic—a standard fourth-order central difference together with a third-order lattice-Boltzmann stencil can be used. It is also possible to use the third-order isotropic stencil E^(6)^ presented in [8, 10]: E^(6)^ has 12 nodes (without the center node) and a span of 2 lattice spacings. The fourth-order central difference together with the D2V17 and E^(6)^ stencils will result in effective stencils of 32 and 24 nodes, respectively; the effective spans are 6 and 4 lattice spacings. In comparison, utilization of (40) requires at least a fourth-order lattice-Boltzmann stencil, for example, D2V37. Note also that it remains to be investigated whether the Lee et al. approach can be extended for the approximation of cross derivatives.

The approximations proposed by Lee et al. [11, 12], Thampi et al. [14], and by Philippi et al. [13] are equal to the approximations presented here in one important sense: expressions for the finite differences are valid regardless of dimension, and any lattice Boltzmann stencil isotropic enough can be utilized; a family of finite differences are defined with single expressions. In contrast, Patra and Karttunen presented more specified isotropic stencils for the numerical computation of second, third, and fourth derivatives [2]. In their approach, the labor of explicitly specifying the coefficients for each case is rewarded with highly compact stencils (a narrow span and a small number of nodes) (sections G and H of [2] appear to involve a mistake: we utilized the coefficients after inverting the signs. Probably the same applies also for section I).

In order to compare the computational efficiency of the aforementioned approaches, we approximate some low- and moderate-rank derivatives with second- and fourth-order accuracy. Our analytical reference function is again given by (47) and a lattice of 600 × 1200 nodes is used. The reference function values are precomputed for the lattice nodes and stored in the computer memory. For all finite-difference stencils, the computational times are measured after repeating the approximations 50 times; that is, the lattice is iterated 50 times and during each iteration the derivatives are approximated once at every node. With this repetition we aim for robust computational times. The additional parameter *ω* now evolves with the iterations: the initial value 1 is incremented by 1/50 after each iteration. The purpose of evolving *ω* is to prevent aggressive compiler optimizations which may compromise the comparisons between different stencils. The reference function values are updated at a node after the approximation of the local derivative.

**Figure 2:**
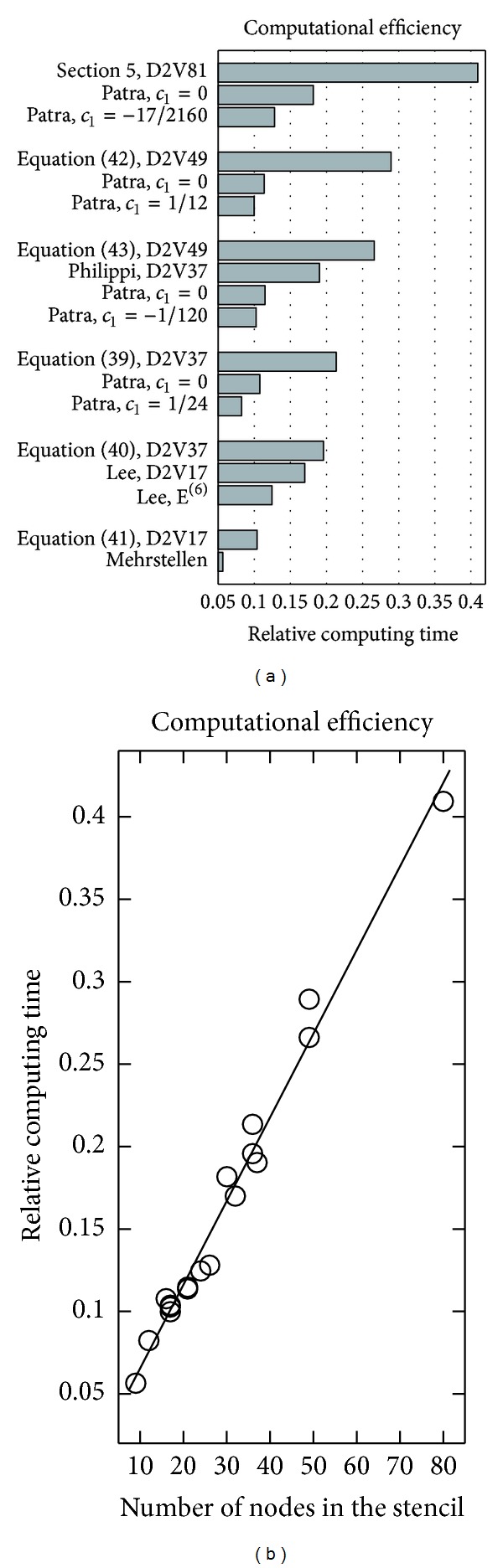
The relative computational times of Table 3 in a graphical format. On the right, the relative times are plotted as functions of the number of nodes in the finite-difference stencils; the line indicates a linear fit. References for the above compared approximations are as follows: Lee et al. [11, 12], Patra and Karttunen [2], and Philippi et al. [13].

The computational times are measured in a computer equipped with *AMD Athlon 64 X2 Dual Core Processor 5000+* (2.6 GHz and 2 GB RAM); the *GNU compiler g++* (version 4.4.1) is used with the option *-O3*. The computational times reported in Table 3 are average values of 5 executions and given in milliseconds. The *Empty load* refers to the Mehrstellen case where only the actual approximation of the derivative is skipped: the relative computational times are defined with respect to the empty load. The relative times are presented also in Figure 2 for visual inspection. With the Patra and Karttunen stencils, we use *c*_2_ = *c*_5_ = 0 in the fourth-order accurate approximations of ∂_*x*_∂^2^. The leading-order error terms related to the isotropic stencils of Patra and Karttunen are (theoretically) independent of the degrees of freedom present. Note that, in the case of odd-rank derivatives, the number of nodes in Table 3 does not agree with the lattice-Boltzmann stencil utilized. This is due to our choice of omitting the zero-velocity vector from the finite-difference stencils on those particular approximations in an attempt to have a fair efficiency comparison between the schemes.

Our first observation is that even the heaviest approximation, utilizing the D2V81 stencil, requires only about 41% more computing time than that of the empty load. In many applications the approximations of derivatives are only a part of the computation: the relative computation times measured suggest that the isotropic derivative approximations do not necessarily introduce a large overhead into the total computing time. Be that as it may, the relative computing times measured appear to be in-line with those reported in [2].

On the other hand, the comparison of relative times between various approximations reveals large differences. In fact, the relative computing times seem to depend linearly on the number of nodes in the finite-difference stencil. The right part of Figure 2 presents a linear fit to the computing times. Furthermore, the stencil with the smallest span among the candidates consistently produces the most accurate approximation. In summary, our observations indicate that compact finite-difference stencils with a small number of nodes are to be preferred. Hence, from strictly a computational efficiency point of view, the approximations proposed in this work are suboptimal and this can be attributed to their generality. At the same time, the generality is also the major asset: it allows a straightforward construction of high-accuracy, isotropic approximations of high-rank derivatives. An interesting compromise between efficiency and generality might be feasible by combining the Philippi et al. approach with the isotropic (nonlattice-Boltzmann) stencils presented in [8–10]—this possibility warrants further investigation.

In our last numerical experiments, we validate the theoretically derived isotropy properties of the finite-difference stencils. This is done by approximating solutions of the diffusion equation ∂_*t*_ *C* = *𝒟*∂^2^ *C* in a two-dimensional case: the local concentration *C* is the dynamic variable and *𝒟* the constant diffusion coefficient. Here we will use *𝒟* = 0.05. For an unbounded domain and when the initial condition is given by a point impulse *P*_*c*_, the analytical solution for the concentration is

(49) $$\begin{array}{c} C\left(r,t\right)=\frac{P_{c}}{4\pi 𝒟t}e^{-r^{2}/4𝒟t}, \end{array}$$

where *r* is the distance from the point impulse. To compute numerical solutions, we approximate the Laplacian with a finite-difference stencil and then treat the resulting ordinary differential equation using the standard second-order *Runge-Kutta scheme* with the *midpoint rule*. We execute the computations on a 141 × 141 grid and the grid spacing is set to *h*_*r*_ = 0.01; the point impulse is located at the center of the domain. The numerical computations are initialized with the analytical field evaluated at time *t*_0_ = 0.01.

**Figure 3:**
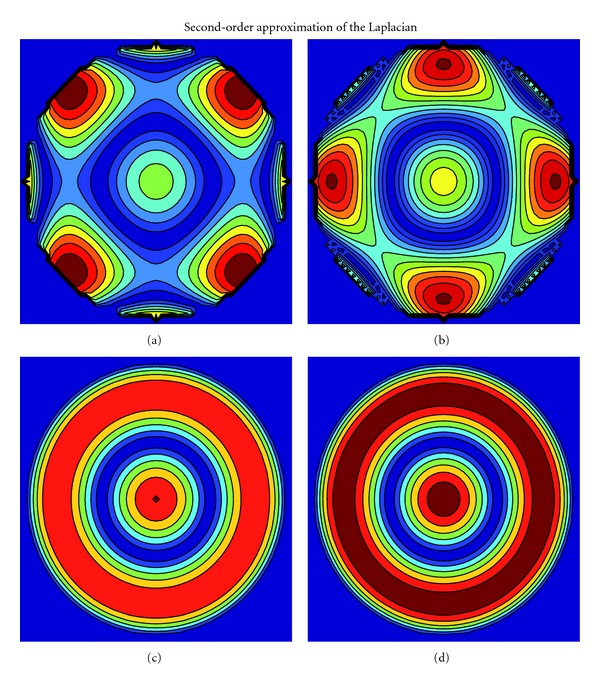
Isocontours of the local relative errors when numerical solutions of the diffusion equation are computed using second-order accurate finite-difference approximations for the Laplacian. The errors are anisotropic for two of the approximations, (a) the standard five-point stencil and (b) (41) with D2Q9, while for the remaining two, (c) the Mehrstellen approximation and (d) (41) with D2V17, the errors are isotropic. The numerical results conform with the theoretical predictions. The colors are not scaled between the approximations.

The numerical solutions are advanced until *t* = 0.1 with a constant time step *δ*_*t*_ = 10^−5^ after which the local relative errors are measured with respect to the analytical solution. Note that the purpose is to investigate the isotropy properties of the finite-difference stencils. Hence, due to the relatively low-order numerical time-integration scheme, the time step has to be small enough in order to allow spatial discretization errors to dominate over the temporal discretization errors. This is especially true for the high-order accurate finite-difference approximations.  Figure 3 presents the local relative errors for four different second-order accurate finite-difference approximations of the Laplacian. For visualization purposes, the local errors are presented only for the domain *r*≲0.3. The errors for two of them, (a) the standard five-point stencil and (b) (41) with D2Q9, are anisotropic while for (c) the Mehrstellen approximation and (d) (41) with D2V17 the errors are isotropic. The local relative errors hence confirm the theoretically predicted isotropy properties.

**Figure 4:**
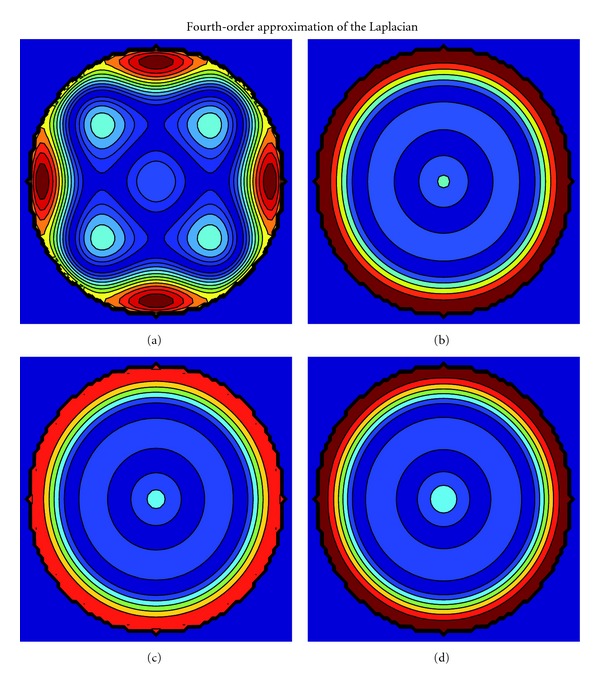
See the caption of Figure 3. The anisotropic error for (a) the standard nine-point stencil and the isotropic errors for (c) (45) with D2V37 and (d) Patra and Karttunen (*c*_1_ = 0) conform with the theoretical predictions. The error for (b) (43) with D2V37 is isotropic: this is not in accordance with the prediction from (37). The colors are not scaled between the approximations.

**Figure 5:**
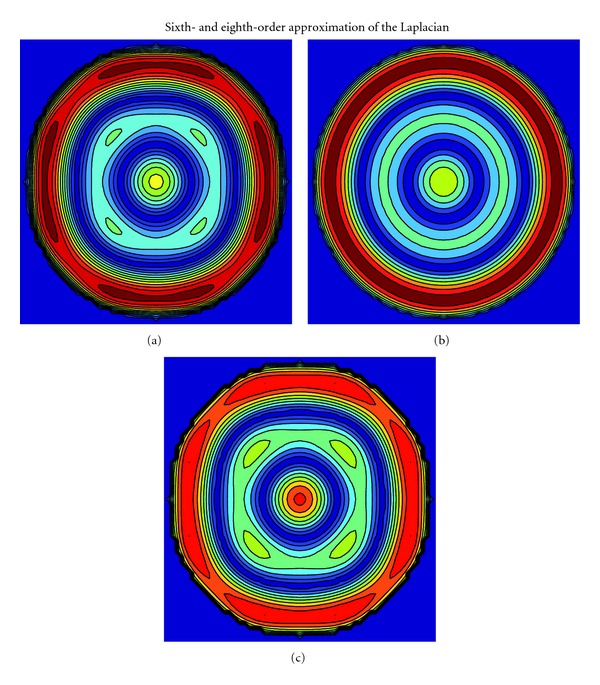
See the caption of Figure 3. The error for the sixth-order approximation for (a) Section 5 with D2V81 is slightly anisotropic while for (b) Section 5 with D2V141 the error is isotropic. The error for the eighth-order approximation for (c) Section 5 with D2V141 also exhibits small anisotropic patterns. The theoretically predicted isotropy properties are hence numerically confirmed. The colors are not scaled between the approximations.

Similarly, Figure 4 presents the local errors for fourth-order accurate finite-difference approximations. For (a) the standard nine-point stencil, (c) (45) with D2V37, and (d) Patra and Karttunen (*c*_1_ = 0) the errors conform with the theoretical predictions. The error for (b) (43) with D2V37 is isotropic: this is not in accordance with the prediction from (37). In general, according to (37), the proposed fourth-order approximation of Laplacian with a fourth-order LB stencil is anisotropic. However, occasionally LB stencils fulfill particular isotropy conditions beyond their order of construction. This can perhaps explain the observed, positive anomaly but, nevertheless, further investigation is needed to explain the observation.  Figure 5 presents the errors for sixth- and eighth-order approximations and, again, the numerical results conform with the theory. In summary, the numerical results verify the theoretically derived isotropy properties, particularly (37), with the above-discussed exception.

## 8. Conclusions

We have presented, and numerically verified, finite differences based on application of lattice-Boltzmann stencils. In particular, high-order accurate isotropic approximations for high-rank derivatives are presented. The expressions for finite differences are valid in arbitrary dimensions, particularly in two and three dimensions, and any lattice Boltzmann stencil isotropic enough can be utilized.

The isotropy and accuracy properties of the proposed approximations are derived directly from the theoretical basis developed in this work. For the numerical verification of the presented theory, we introduced 5th-, 6th-, and 8th-order two-dimensional lattice-Boltzmann stencils. Moreover, in the construction of the finite differences, we extended the theory of Hermite polynomial tensors in discrete spaces. First, we proved the equivalency between two approaches for constructing lattice-Boltzmann stencils. Secondly, we presented the expressions for discrete inner products between monomials and Hermite polynomial tensors. These inner products can be useful tools, for example, in the Chapman-Enskog analysis of lattice-Boltzmann schemes and, more generally, in any numerical analysis involving functions expanded in Hermite polynomial tensors.

Finally, the isotropic finite-difference approximations appear to be more stable than their anisotropic counterparts. Hence, an analytical and numerical investigation on stability properties of isotropic finite differences is a relevant research topic for the future.

## Figures and Tables

**Table 1:** Specification of a fifth-order lattice-Boltzmann stencil: *a*_*s*_ = 1.148732248838539. The second column indicates the number of velocity vectors, obtained by permutating the vector components, sharing the given weight.

**c**_*i*_^⋆^	*p*	*w*_*i*_ (D2V49)
(0, 0)	1	2.1486215695621588 × 10^−1^
(±1, 0)	4	1.0466324937773665 × 10^−1^
(±1, ±1)	4	5.9279902836032117 × 10^−2^
(±2, 0)	4	1.6329411241508002 × 10^−2^
(±2, ±1)	8	6.6560728515645176 × 10^−3^
(±2, ±2)	4	1.4236189699711052 × 10^−3^
(±3, 0)	4	3.8641376938724455 × 10^−4^
(±3, ±1)	8	4.4284732290059492 × 10^−4^
(±3, ±3)	4	2.9169039140666429 × 10^−6^
(±5, 0)	4	8.8685055032561952 × 10^−7^
(±4, ±4)	4	2.2046291628739507 × 10^−7^

**Table 2:** Specification of a sixth-order (D2V81) and eighth-order (D2V141) lattice-Boltzmann stencil: *a*_*s*_ = 0.970008498739395 and *a*_*s*_ = 0.8369204054303525, respectively. The second column indicates the number of velocity vectors, obtained by permutating the vector components, sharing the given weight.

**c**_*i*_^⋆^	*p*	*w*_*i*_ (D2V81)	*w*_*i*_ (D2V141)
(0, 0)	1	1.5381368561993594 × 10^−1^	1.1508949125706189 × 10^−1^
(±1, 0)	4	9.0384577224028734 × 10^−2^	7.5595334899625166 × 10^−2^
(±1, ±1)	4	6.0915016936039915 × 10^−2^	5.7734363121950370 × 10^−2^
(±2, 0)	4	2.4220944974511140 × 10^−2^	2.9033095241565582 × 10^−2^
(±2, ±1)	8	1.3146486542343966 × 10^−2^	1.8074672747613353 × 10^−2^
(±2, ±2)	4	3.9625069477690709 × 10^−3^	7.4435479269746455 × 10^−3^
(±3, 0)	4	1.8542634450574055 × 10^−3^	4.2549928887228462 × 10^−3^
(±3, ±1)	8	1.6035095499280431 × 10^−3^	3.7759145451527918 × 10^−3^
(±3, ±2)	8	2.2249142091156890 × 10^−4^	9.5510661516062627 × 10^−4^
(±4, 0)	4	1.0005397521769796 × 10^−4^	4.9926973701248283 × 10^−4^
(±4, ±1)	8	3.4398327658312326 × 10^−5^	2.1766901272228135 × 10^−4^
(±3, ±3)	4	5.4494804327695976 × 10^−5^	2.7427360033189315 × 10^−4^
(±4, ±2)	8	1.8837067344235765 × 10^−5^	1.3852638332696371 × 10^−4^
(±5, 0)	4	2.4044498210968849 × 10^−6^	1.2905073342509215 × 10^−5^
(±4, ±3)	8	—	5.9323648903820310 × 10^−6^
(±5, ±1)	8	—	1.6161185887309810 × 10^−5^
(±5, ±2)	8	4.3501093550344725 × 10^−7^	2.4159493337948245 × 10^−6^
(±4, ±4)	4	—	3.3101853527875648 × 10^−6^
(±5, ±3)	8	—	1.3086410701892049 × 10^−6^
(±6, 0)	4	—	1.1118416570950374 × 10^−7^
(±6, ±1)	8	—	4.6121305137932601 × 10^−7^
(±6, ±3)	8	—	3.6530518727364592 × 10^−8^
(±7, 0)	4	—	8.3273853753395782 × 10^−9^
(±7, ±2)	8	—	2.3109247814347261 × 10^−9^

**Table 3:** Comparison of various approximations for low- and moderate-rank derivatives with second- and fourth-order accuracy. The computational times are given in milliseconds and the * Empty load* refers to the Mehrstellen case where only the actual approximation is skipped: the relative computational times are defined with respect to the empty load. References for the below compared approximations are as follows: Lee and Lin [11], Lee and Fischer [12], Patra and Karttunen [2], and Philippi et al. [13].

	Nodes	Span	C. time	Rel. time	Rel. *L*_2_-error
Empty load	—	—	7984	0.0	—

∂^ 2^ (2nd-order approximation)					
Mehrstellen	9	1	8435	0.0565	4.124 × 10^−5^
Equation (41), D2V17	17	3	8813	0.1038	9.160 × 10^−5^

∂_*x*_ (4th-order approximation)					
Lee, E^(6)^	24	4	8978	0.1245	1.632 × 10^−8^
Lee, D2V17	32	6	9341	0.1699	1.678 × 10^−8^
Equation (40), D2V37	36	3	9547	0.1958	1.491 × 10^−8^

∂_*x*_∂^ 2^ (2nd-order approximation)					
Patra, *c*_1_ = 1/24	12	2	8641	0.0823	1.237 × 10^−4^
Patra, *c*_1_ = 0	16	2	8844	0.1077	1.237 × 10^−4^
Equation (39), D2V37	36	3	9688	0.2134	1.727 × 10^−4^

∂^ 2^ (4th-order approximation)					
Patra, *c*_1_ = −1/120	17	2	8803	0.1026	2.721 × 10^−9^
Patra, *c*_1_ = 0	21	2	8900	0.1147	2.721 × 10^−9^
Philippi, D2V37	37	3	9503	0.1903	4.969 × 10^−9^
Equation (43), D2V49	49	5	10109	0.2662	1.758 × 10^−8^

∂^ 2^∂^ 2^ (2nd-order approximation)					
Patra, *c*_1_ = 1/12	17	2	8781	0.0998	8.247 × 10^−5^
Patra, *c*_1_ = 0	21	2	8891	0.1136	8.248 × 10^−5^
Equation (42), D2V49	49	5	10294	0.2893	1.875 × 10^−4^

∂_*x*_∂^ 2^ (4th-order approximation)					
Patra, *c*_1_ = −17/2160	26	3	9006	0.1281	1.428 × 10^−8^
Patra, *c*_1_ = 0	30	3	9435	0.1817	1.428 × 10^−8^
Section 5, D2V81	80	5	11253	0.4095	3.456 × 10^−8^

## Appendices

### A. Hermite Polynomial Tensors and Monomials

Using the notation presented in Section 2.1, the explicit expressions for the first few Hermite polynomial tensors are **H** ^(0)^ = 1 = **M** ^(0)^, **H** ^(1)^ = **v** = **M** ^(1)^, and

(A.1) $$\begin{array}{c} H^{\left(2\right)}=M^{\left(2\right)}-\Delta^{\left(1\right)},\\ H^{\left(3\right)}=M^{\left(3\right)}-M^{\left(1\right)}⊛\Delta^{\left(1\right)},\\ H^{\left(4\right)}=M^{\left(4\right)}-M^{\left(2\right)}⊛\Delta^{\left(1\right)}+\Delta^{\left(2\right)},\\ H^{\left(5\right)}=M^{\left(5\right)}-M^{\left(3\right)}⊛\Delta^{\left(1\right)}+M^{\left(1\right)}⊛\Delta^{\left(2\right)},\\ H^{\left(6\right)}=M^{\left(6\right)}-M^{\left(4\right)}⊛\Delta^{\left(1\right)}+M^{\left(2\right)}⊛\Delta^{\left(2\right)}-\Delta^{\left(3\right)},\\ H^{\left(7\right)}=M^{\left(7\right)}-M^{\left(5\right)}⊛\Delta^{\left(1\right)}+M^{\left(3\right)}⊛\Delta^{\left(2\right)}-M^{\left(1\right)}⊛\Delta^{\left(3\right)},\\ H^{\left(8\right)}=M^{\left(8\right)}-M^{\left(6\right)}⊛\Delta^{\left(1\right)}+M^{\left(4\right)}⊛\Delta^{\left(2\right)}-M^{\left(2\right)}⊛\Delta^{\left(3\right)}+\Delta^{\left(4\right)}. \end{array}$$

Note that, for example, in the last expression above the second, third, and fourth terms at the right-hand side involve summation of 28, 70, and 28 tensor products, respectively. Furthermore, the expressions for even and odd rank Hermite polynomial tensors involve only even and odd rank monomials, respectively.

In a similar way, monomials can be expressed as linear combinations of Hermite polynomial tensors. By using the explicit expressions for the Hermite polynomial tensors presented above, the first few monomials can be written as

(A.2) $$\begin{array}{c} M^{\left(2\right)}=H^{\left(2\right)}+\Delta^{\left(1\right)},\\ M^{\left(3\right)}=H^{\left(3\right)}+H^{\left(1\right)}⊛\Delta^{\left(1\right)},\\ M^{\left(4\right)}=H^{\left(4\right)}+H^{\left(2\right)}⊛\Delta^{\left(1\right)}+\Delta^{\left(2\right)},\\ M^{\left(5\right)}=H^{\left(5\right)}+H^{\left(3\right)}⊛\Delta^{\left(1\right)}+H^{\left(1\right)}⊛\Delta^{\left(2\right)},\\ M^{\left(6\right)}=H^{\left(6\right)}+H^{\left(4\right)}⊛\Delta^{\left(1\right)}+H^{\left(2\right)}⊛\Delta^{\left(2\right)}+\Delta^{\left(3\right)},\\ M^{\left(7\right)}=H^{\left(7\right)}+H^{\left(5\right)}⊛\Delta^{\left(1\right)}+H^{\left(3\right)}⊛\Delta^{\left(2\right)}+H^{\left(1\right)}⊛\Delta^{\left(3\right)},\\ M^{\left(8\right)}=H^{\left(8\right)}+H^{\left(6\right)}⊛\Delta^{\left(1\right)}+H^{\left(4\right)}⊛\Delta^{\left(2\right)}+H^{\left(2\right)}⊛\Delta^{\left(3\right)}+\Delta^{\left(4\right)}. \end{array}$$

Above we have used the identities Δ^(1)^⊛Δ^(1)^ = 2Δ^(2)^, Δ^(1)^⊛Δ^(2)^ = 3Δ^(3)^, and Δ^(1)^⊛Δ^(3)^ = 4Δ^(4)^ repeatedly in the derivation. The expressions for even and odd rank monomials include only even and odd rank Hermite polynomial tensors, respectively.

### B. Proof of Equivalency between Quadrature Problems

We prove that a solution to either of the two quadrature problems, (17) or (18), will also be solution to the other one (up to the same order). First we assume a solution *w*_*i*_ and **v**_*i*_ for *i* = 0,…, *q* − 1, obtained by solving the system of equations arising from (18). That is, (18) holds for *n* ≤ *N*, where *N* is the given order. Next we write the tensor product of monomials in terms of Hermite polynomial tensors in a specific way:

(B.1) M(n)M(n)=bn(0)H(n)H(n)+bn−1(2)H(n−1)H(n−1)+⋯ +b0(2n)H(0)H(0),

where the polynomial tensor coefficients **b**_*l*_^(*k*)^ are left undetermined. Then we substitute this expression into the right-hand side of (17) and obtain

(B.2) ∫ω(v)M(n)M(n)dv=∫ω(v)(bn(0)H(n)H(n)+⋯ +b0(2n)H(0)H(0))dv.

With the solution for (18), we can equate the right-handside of the above equation to

(B.3) bn(0)∑iwiHi(n)Hi(n)+⋯+b0(2n)∑iwiHi(0)Hi(0)  =∑iwi(bn(0)Hi(n)Hi(n)+⋯+b0(2n)Hi(0)Hi(0)).

Because expression (B.1) is true for every **v**, it is especially true for **v**_*i*_. Hence, we can use expression (B.1) in (B.3) to obtain the left-hand side of (17) which concludes the first half of our proof. In other words, the solution to (18) will also satisfy (17) for *n* ≤ *N*; that is, the inner products of the monomials in discrete and continuous space will be also matched. In the second half of the proof it is shown that the solution to (17) will also satisfy (18). The proof proceeds in exactly the same way as the first half above and, hence, is not shown here.

### C. Proof of the Summation Formula for the Monomials

We use induction to prove (20). The expansion is valid for **M** ^(0)^ and **M** ^(1)^. Then, by first using the induction assumption and (15),

(C.1) Mpq(n+1)=vpMq(n)=vp(Hq(n)+Hq(n−2)⊛Δq(1)+Hq(n−4)⊛Δq(2)+⋯)=(Hpq(n+1)+Δpq(1)⊛Hq(n−1)) +(Hpq(n−1)+Δpq(1)⊛Hq(n−3))⊛Δq(1)     +(Hpq(n−3)+Δpq(1)⊛Hq(n−5))⊛Δq(2)+⋯.

By rearranging the terms on the right-hand side we get

(C.2) Hpq(n+1)+(Δpq(1)⊛Hq(n−1)+Hpq(n−1)⊛Δq(1))   +(Δpq(1)⊛Δq(1)⊛Hq(n−3)+Hpq(n−3)⊛Δq(2))   +Δpq(1)⊛Δq(2)⊛Hq(n−5)+⋯

which, after applying (6), becomes

(C.3) Hpq(n+1)+(Δpq(1)⊛Hq(n−1)+Hpq(n−1)⊛Δq(1))   +(Δpq(2)⊛Hq(n−3)+Hpq(n−3)⊛Δq(2))   +Δpq(3)⊛Hq(n−5)+⋯.

Finally, we use (10) and arrive at

(C.4) Mpq(n+1)=Hpq(n+1)+(H(n−1)⊛Δ(1))pq +(H(n−3)⊛Δ(2))pq+⋯.

The expansion terminates properly, and hence we have completed the induction.
